# Vasoprotective Effects of Urocortin 1 against Atherosclerosis *In Vitro* and *In Vivo*

**DOI:** 10.1371/journal.pone.0110866

**Published:** 2014-12-02

**Authors:** Akinori Hasegawa, Kengo Sato, Remina Shirai, Rena Watanabe, Keigo Yamamoto, Kaho Watanabe, Kyoko Nohtomi, Tsutomu Hirano, Takuya Watanabe

**Affiliations:** 1 Laboratory of Cardiovascular Medicine, Tokyo University of Pharmacy and Life Sciences, Tokyo, Japan; 2 Department of Medicine, Division of Diabetes, Metabolism, and Endocrinology, Showa University School of Medicine, Tokyo, Japan; University of Sassari, Italy

## Abstract

**Aim:**

Atherosclerosis is the complex lesion that consists of endothelial inflammation, macrophage foam cell formation, vascular smooth muscle cell (VSMC) migration and proliferation, and extracellular matrix production. Human urocortin 1 (Ucn1), a 40-amino acid peptide member of the corticotrophin-releasing factor/urotensin I family, has potent cardiovascular protective effects. This peptide induces potent and long-lasting hypotension and coronary vasodilation. However, the relationship of Ucn1 with atherosclerosis remains unclear. The present study was performed to clarify the effects of Ucn1 on atherosclerosis.

**Methods:**

We assessed the effects of Ucn1 on the inflammatory response and proliferation of human endothelial cells (ECs), human macrophage foam cell formation, migration and proliferation of human VSMCs, extracellular matrix expression in VSMCs, and the development of atherosclerosis in apolipoprotein E-deficient (*Apoe* ^−/−^) mice.

**Results:**

Ucn1 significantly suppressed cell proliferation without inducing apoptosis, and lipopolysaccharide-induced up-regulation of monocyte chemoattractant protein-1 and intercellular adhesion molecule-1 in human ECs. Ucn1 significantly reduced oxidized low-density lipoprotein-induced foam cell formation with a significant down-regulation of CD36 and acyl-CoA:cholesterol acyltransferase 1 in human monocyte-derived macrophages. Ucn1 significantly suppressed the migration and proliferation of human VSMCs and increased the activities of matrix metalloproteinase-2 (MMP2) and MMP9 in human VSMCs. Intraperitoneal injection of Ucn1 into *Apoe* ^−/−^ mice for 4 weeks significantly retarded the development of aortic atherosclerotic lesions.

**Conclusions:**

This study provided the first evidence that Ucn1 prevents the development of atherosclerosis by suppressing EC inflammatory response and proliferation, macrophage foam cell formation, and VSMC migration and proliferation. Thus, Ucn1 could serve as a novel therapeutic target for atherosclerotic cardiovascular diseases.

## Introduction

Atherosclerosis is a chronic inflammatory response to the injury in the arterial wall [1]. Endothelial inflammation is characterized by increased production of pro-atherogenic molecules and inflammatory cytokines such as interleukin-6 (IL6), monocyte chemoattractant protein-1 (MCP1), intercellular adhesion molecule-1 (ICAM1), and E-selectin in endothelial cells (ECs), and monocyte adhesion and infiltration into the neointima lesion, followed by oxidized low-density lipoprotein (oxLDL)-induced transformation of macrophages into foam cells [2]. Accumulation of cholesterol ester (CE) in macrophages is a hallmark of foam cell formation [2]. This accumulation depends on the balance between the uptake of oxLDL *via* CD36 and the efflux of free cholesterol (FC) controlled by ATP-binding cassette transporter A1 (ABCA1) [2]. To protect the cells from the toxicity that would result from excessive FC accumulation, the FC is esterified to CE by acyl-CoA:cholesterol acyltransferase-1 (ACAT1) [2]. Apart from accumulation of macrophage foam cells, the migration and proliferation of vascular smooth muscle cells (VSMCs), EC proliferation, and the production of extracellular matrix (ECM) components, such as collagens, matrix metalloproteinases (MMPs), fibronectin, and elastin, contribute to the progression of atherosclerotic plaques [1], [3].

Urocortin 1 (Ucn1), a 40-amino-acid peptide related to the corticotrophin-releasing factor (CRF)/urotensin I family, was originally cloned from rat and thereafter the human brain [4]. In the cardiovascular system, Ucn1 and its receptors, CRF-R1 and CRF-R2, are expressed in cardiomyocytes, ECs, VSMCs, and macrophages [5]–[7]. Both animal and human studies have shown that Ucn1 is released when the heart is under stress, such as ischemia or heart failure [8], [9]. Secretion of Ucn1 is stimulated by reactive oxygen species (ROS), angiotensin II (AngII), lipopolysaccharide (LPS), and inflammatory cytokines, such as IL6, interferon-γ, and tumor necrosis factor-α (TNFα) [10], [11]. Thereby, Ucn1 exerts cardioprotective effects, such as causing coronary vasodilatation, positive inotropic effect, and an anti-apoptotic effect in the myocardium after ischemia-reperfusion injury [8], [12]. In clinical practice, plasma Ucn1 levels are elevated in patients with acute myocardial infarction or heart failure [13], [14]. A genomics array analysis highlighted Ucn1 as a favorable molecule for cardiovascular diseases [15]. However, the direct association between Ucn1 and atherogenesis has not yet been reported.

In the present study, we assessed the suppressive effects of Ucn1 on the inflammatory response and proliferation of human ECs, human macrophage foam cell formation, the migration, proliferation, and ECM production in human VSMCs *in vitro*, and the development of atherosclerotic lesions in apolipoprotein E-deficient (*Apoe* ^−/−^) mice, an animal model of atherosclerosis, *in vivo*.

## Materials and Methods

### Human Cell Culture

This investigation was approved by the Ethics Committee of Tokyo University of Pharmacy and Life Sciences. Written informed consent was obtained from 15 healthy volunteers (7 men, 8 women; aged 19–22) who were free of hypertension, diabetes, dyslipidemia, and arteriosclerotic vascular diseases and were taking no medications. Human peripheral mononuclear cells were isolated from their blood. Monocytes purified using anti-CD14 antibody-conjugated magnetic microbeads (Miltenyi Biotec, Auburn, CA) were seeded onto 3.5-cm dishes (1×10^6^ cells/1 ml/dish) for cholesterol esterification assay and immunoblotting analysis [16]–[19]. Cells were incubated at 37°C in 5% CO_2_ for 7 days in RPMI-1640 medium supplemented with 10% human serum, 0.05 mg/ml streptomycin, 50 U/ml penicillin, and the indicated concentrations of human Ucn1 (Abgent, San Diego, CA). The medium in each dish was replaced with fresh medium containing Ucn1 every 3 days.

### Cholesterol Esterification Assay

Human macrophages differentiated by 7-day culture with the indicated concentrations of Ucn1 were incubated for 19 h with 50 µg/ml human oxLDL in the presence of 0.1 mmol/l [^3^H]oleate (PerkinElmer, Yokohama, Japan) conjugated with bovine serum albumin [16]. Cellular lipids were extracted and the radioactivity of cholesterol-[^3^H]oleate was determined by thin-layer chromatography.

### Migration Assay

Human aortic smooth muscle cells (HASMCs; Lonza, Walkersville, MD) at passage 7 were seeded onto 3.5-cm dishes (5×10^4^ cells/1 ml/dish). Cells were incubated at 37°C in 5% CO_2_ for 7 h in smooth muscle cell basal medium (SmBM; Lonza) supplemented with 0.5 ng/ml human epidermal growth factor, 5 µg/ml insulin, 2 ng/ml human fibroblast growth factor, 50 µg/ml gentamicin, 50 ng/ml amphotericin B, and 5% fetal bovine serum (FBS). Subsequently, while HASMCs were incubated in serum-free SmBM with or without the indicated concentrations of AngII (Sigma, St. Louis, MO) and/or Ucn1 for 5 h, photographs of cells were taken at 10-min intervals. The average migration distance of 10 cells randomly selected in each dish was measured using a BIOREVO BZ-9000 microscope (Keyence, Osaka, Japan) [16].

### Proliferation Assay

HASMCs or human EA.hy926 ECs at passage 5–10 were seeded onto 96-well plates (1×10^4^ cells/100 µl/well) and incubated at 37°C in 5% CO_2_ for 24 h in SmBM supplemented with 5% FBS and above additives or Dulbecco's modified Eagle's medium (DMEM) supplemented with 5% FBS, 4.5 mg/ml d-glucose, 0.584 mg/ml l-glutamine, 0.05 mg/ml streptomycin, and 50 U/ml penicillin G, respectively. Cells were incubated for 48 h with the indicated concentrations of Ucn1 with renewal of each medium. Subsequently, 10 µl of WST-8 solution (Cell Count Reagent SF; Nacalai Tesque, Kyoto, Japan) was added to each well [16]. After 1 h of incubation, the amount of formazan product was determined by measuring the absorbance at 450 nm using a Sunrise Remote R-micro plate reader (Tecan, Kawasaki, Japan).

### Immunocytochemistry

HASMCs or human EA.hy926 ECs were seeded onto 12-well plates (1×10^5^ cells/1 ml/well) and incubated at 37°C in 5% CO_2_ for 24 h in the same conditioning medium, followed by 48 h-incubation with the indicated concentrations of Ucn1. Cells were fixed with 4% paraformaldehyde in phosphate-buffered saline (PBS) and stained with rabbit polyclonal anti-Ki-67 antibody (Leica Biosystems, Newcastle upon Tyne, UK), followed by anti-rabbit Alexa Fluor 488 (Life Technologies, Carlsbad, CA). Terminal deoxynucleotidyl transferase- mediated deoxyuridine triphosphate-biotin nick end labelling (TUNEL) staining was performed using an In Situ Apoptosis Detection Kit (Takara Bio, Otsu, Japan). Nuclei were visualized by 4′,6-diamidino-2-phenylindole (DAPI) staining. All samples were mounted with Fluorescent Mounting Medium (Dako, Glostrup, Denmark). Fluorescence-stained cells were examined on confocal microscope (FV1000D, Olympus, Tokyo, Japan). Fluorescence was detected with wavelengths for excitation at 488 nm (Alexa Fluor 488) and 360 nm (DAPI).

### Western Blotting

Aliquots of 20 µg of protein extracts from human macrophages and HASMCs were separated by 10% sodium dodecyl sulfate-polyacrylamide gel electrophoresis (SDS-PAGE) and subjected to immunoblotting with the following antibodies: CD36 (R&D Systems, Minneapolis, MN), ACAT1 (Santa Cruz Biotechnology, Santa Cruz, CA), ABCA1, collagen-1 (Novus Biologicals, Littleton, CO), collagen-3, fibronectin, α-tubulin, MMP2 (GeneTex, Irvine, CA), MMP9 (EnoGene, Atlanta, GA), elastin (Bioss, Woburn, MA), or β-actin (Sigma) [16]–[19]. The densities of the bands were measured using a Densitograph System (Ez-Capture II and CS Analyzer 3.0, ATTO, Tokyo, Japan).

### Zymography

The activities of MMP2 and MMP9 were determined using a gelatin-zymography kit (Cosmo Bio, Tokyo, Japan) [20]. The culture supernatants of HASMCs (15 µg) were mixed with 5 µl of sample buffer and fractionated by 10% SDS-PAGE using 0.1% gelatin. After electrophoresis, the gel was washed with renaturing buffer (2% Triton X-100) for 1 h. The gel was incubated for 20 h at 37°C in a reaction buffer (1% Triton X-100) and then stained with Coomassie brilliant blue. The densities of the bands were measured using an image analyzer (NIH ImageJ, Bethesda, MD).

### Reverse Transcription-Polymerase Chain Reaction (RT-PCR)

Human umbilical vein endothelial cells (HUVECs; Kurabo, Osaka, Japan) were pre-treated with or without an indicated concentration of Ucn1 in HuMedia-EG2 medium (Kurabo) for 30 min, and further incubated with Ucn1+LPS (1 µg/ml) for 2 h. Total RNA was extracted using a High Pure RNA Isolation Kit (Roche Diagnostics, Mannheim, Germany) according to the manufacturer's instructions. Complementary DNAs were synthesized from isolated RNA templates with a High Capacity cDNA Reverse Transcription Kit (Applied Biosystems, Foster City, CA). The mRNAs of IL6, MCP1, ICAM1, E-selectin, and glyceraldehyde-3-dehydrogenase (GAPDH) in 50 ng of each sample were detected by RT-PCR, using GoTaq Green Master Mix (Promega, Madison, WI). The sequence of the primers and product size are listed in Table 1. The PCR products were shown by 2% agarose gel electrophoresis, and densitometric analyses were performed as described above.

**Table 1:** Primer Sequences Used for RT-PCR.

Gene		Primer Sequence (5′→3′)	Product Size (bp)
IL6	Forward	ATGAACTCCTTCTCCACAAGCGC	628
	Reverse	GAAGAGCCCTCAGGCTGGACT	
MCP1	Forward	CAATAGGAAGATCTCAGTGC	189
	Reverse	GTGTTCAAGTCTTCGGAGTT	
ICAM1	Forward	CGACTGGACGACAGGGATTGT	290
	Reverse	ATTATGACTGCGGCTGCTACC	
E-Selectin	Forward	CCTACAAGTCCTCTTGTGCCTTC	206
	Reverse	ACAGGCGAACTTGCACACA	
GAPDH	Forward	ACCACAGTCCATGCCATCAC	451
	Reverse	TCCACCACCCTGTTGCTGTA	

IL6 = interleukin-6, MCP1 = monocyte chemoattractant protein-1, ICAM1 = intercellular adhesion molecule-1, GAPDH = glyceraldehyde-3-dehydrogenase.

### Animal Experiments

Animal experiments were performed in accordance with the NIH Guidelines for the Care and Use of Laboratory Animals and were approved by the Institutional Animal Care and Use Committee of Tokyo University of Pharmacy and Life Sciences. A total of 19 male spontaneously hyperlipidemic *Apoe* ^−/−^ mice (C57BL/6. KOR/StmSlc-*Apoe^shl^* mice) at the age of 9 weeks were purchased from Japan SLC Inc. (Hamamatsu, Japan) and kept on a normal diet until the age of 13 weeks. Subsequently, a high cholesterol diet (Oriental Yeast, Tokyo, Japan) was started [16]. At 17 weeks of age, 3 mice were sacrificed as a control before injection. The remaining 16 mice were divided into 2 groups; 7 mice and 9 mice, were intraperitoneally injected once daily for 4 weeks with saline (vehicle) or Ucn1 (64 nmol/kg/day; Abgent), respectively. The doses of Ucn1 and its administration methods were decided on the basis of our preliminary examinations.

### Animal Measurements

Body weight and food intake were measured during a protocol. Systolic and diastolic blood pressures were measured using the indirect tail-cuff method (Kent Scientific, Torrington, CT). Blood samples were collected after a 4-h fast. Plasma concentrations of glucose and total cholesterol were measured by enzymatic methods [16]. Plasma Ucn1 concentration was measured by enzyme-linked immunosorbent assay (ELISA Kit for Ucn1, Uscn Life Science, Houston, TX).

### Atherosclerotic Lesion Assessment

After 4 weeks of injection, the *Apoe* ^−/−^ mice were anesthetized with diethyl ether. The whole aorta was washed by perfusion with PBS, and fixed with 4% formaldehyde. The aorta was excised from the aortic sinus to the abdominal area and the connective and adipose tissues were carefully removed. The entire aorta and cross-sections of the aortic sinus were stained with oil red O for assessment of atherosclerotic lesions [16], [17], [21]. Monocyte/macrophage and VSMC contens in the atherosclerotic lesions were visualized by staining with anti-MOMA-2 antibody (Millipore, Billerica, MA) or anti-α-smooth muscle actin (SMA) antibody (Sigma), respectively [16]. These areas of the aortic wall were traced by an investigator blind to the treatment and measured as described previously [16], [17], [21].

### Statistical Analysis

All values are expressed as means ± SEM. The data were compared by unpaired Student's *t* test between 2 groups and 1-way ANOVA followed by Bonferroni's post hoc test among ≥3 groups using Statview-J 5.0 (SAS Institute, Cary, NC). A value of *P*<0.05 was considered to be statistically significant.

## Results

### Effects of Ucn1 on Inflammatory Response and Cell Proliferation in Human ECs

**Figure 1:**
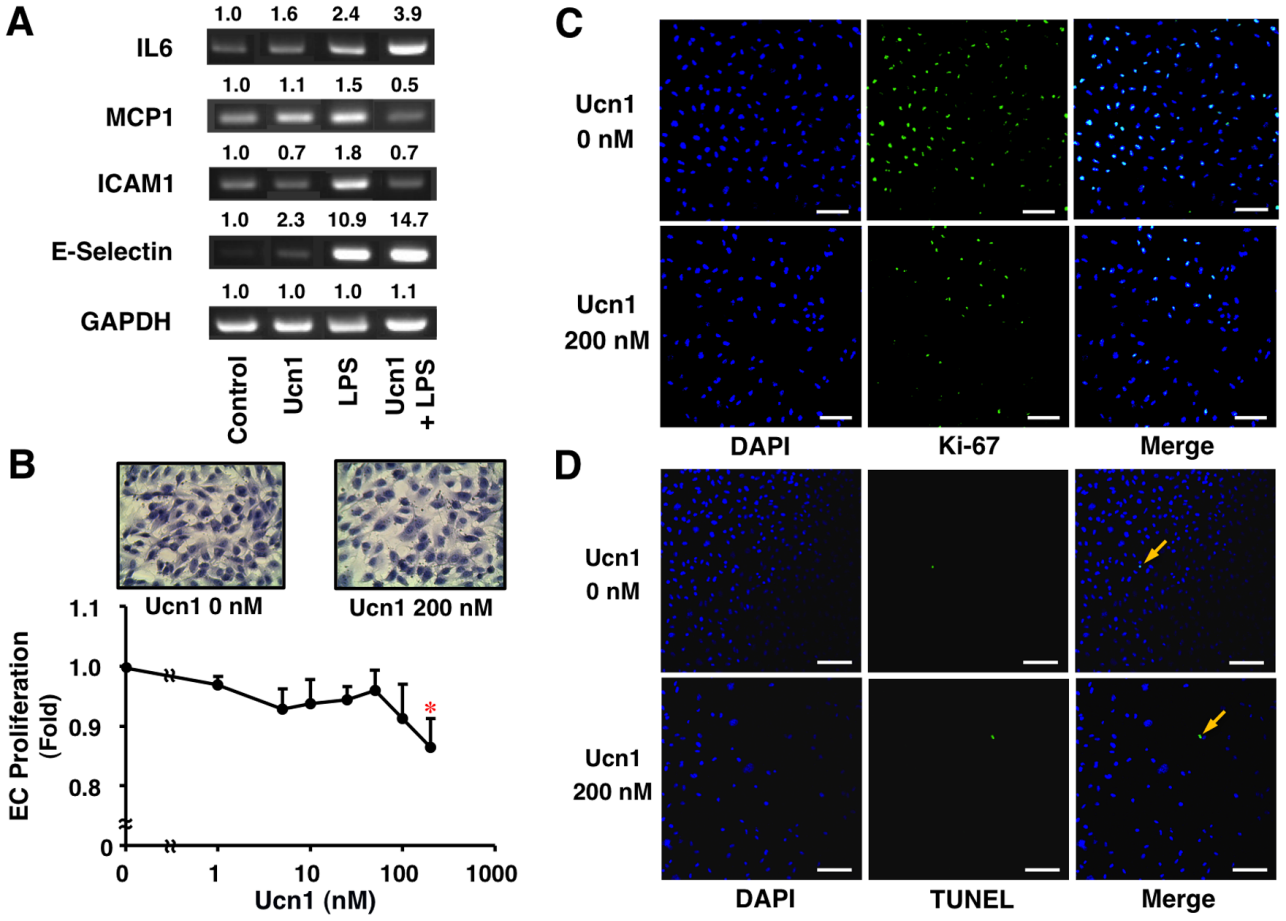
Suppressive effects of Ucn1 on inflammatory response and cell proliferation in human ECs. (A) HUVECS were pre-treated with or without Ucn1 (200 nmol/l) for 30 min and then incubated with Ucn1 (200 nmol/l)+LPS (1 µg/ml) for 2 h. The mRNA expressions of IL6, MCP1, ICAM1, and E-selectin were analyzed by RT-PCR. GAPDH served as a loading control. Data are representative of 2 independent experiments. (B) Proliferation of EA.hy926 cells was determined by WST-8 assay after 48-h incubation in conditioning medium with the indicated concentrations of Ucn1. Data are expressed as means ± SEM from 4 independent experiments. *P<0.05 vs. 0 nmol/l of Ucn1. (B–D) EA.hy926 cells were stained with Hematoxylin Eosine, anti-Ki-67 antibody, and TUNEL method. DAPI was used to stain the nucleus. Representative images are shown. Scale bar = 100 µm.

Treatment with LPS (1 µg/ml) obviously accelerated the mRNA expressions of IL6, MCP1, ICAM1, and E-selectin in HUVECs compared with control (Fig. 1A). Ucn1 (200 nmol/l) suppressed the LPS-induced up-regulations of MCP1 and ICAM1, but potentiated those of IL6 and E-selectin at mRNA levels (Fig. 1A).

Ucn1 significantly suppressed the proliferation of EA.hy926 cells in a concentration-dependent manner (*P*<0.05; Fig. 1B). The maximum inhibitory effect observed at 200 nmol/l of Ucn1 on cell proliferation was confirmed by the reductions of cell number (Fig. 1B) and Ki-67 expression, a proliferation cell cycle marker (Fig. 1C). However, the induction of apoptosis was not observed at 200 nmol/l of Ucn1 (Fig. 1D).

### Effects of Ucn1 on Human Macrophage Foam Cell Formation

**Figure 2:**
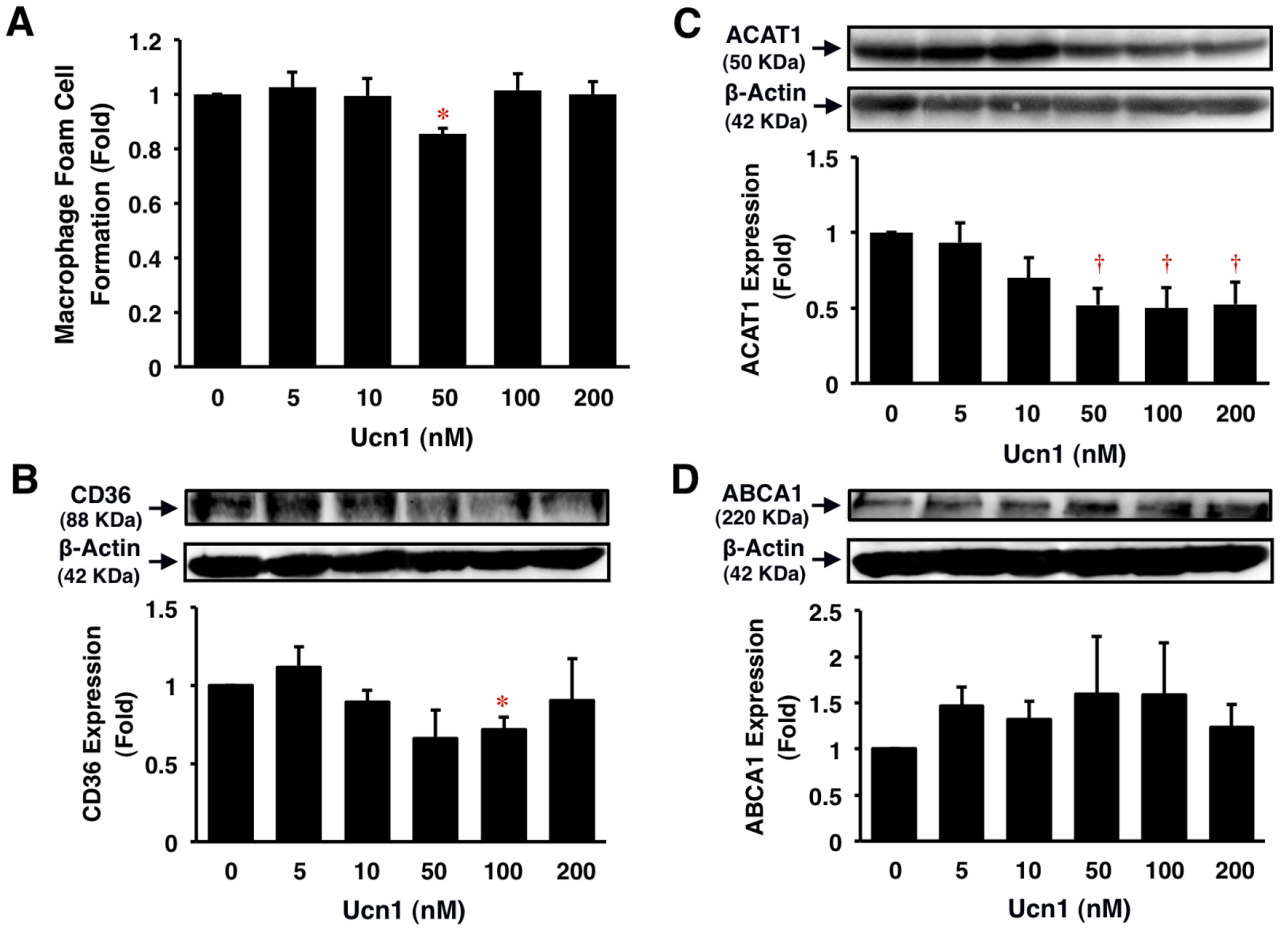
Suppressive effects of Ucn1 on foam cell formation and related protein expression in human monocyte-derived macrophages. Human monocytes were incubated for 7 days with RPMI-1640 supplemented with 10% human serum and the indicated concentrations of Ucn1, followed by a 19 h-incubation with 50 µg/ml oxLDL in the presence of 0.1 mmol/l [^3^H]oleate. Intracellular CE accumulation was determined from the radioactivity of cholesterol-[^3^H]oleate. Otherwise, before the addition of oxLDL, cells were harvested and subjected to immunoblotting analyses for CD36, ACAT1, or ABCA1. β-Actin served as a loading control. Data are expressed as means ± SEM from 4–6 independent experiments with monocytes from 4–6 different donors. Baseline (1 fold) = 8.4±2.2 nmol/mg cell protein. *P<0.05, **^†^** *P*<0.001 vs. 0 nmol/l of Ucn1.

Ucn1 significantly suppressed oxLDL-induced CE accumulation by 20% at 50 nmol/l in human monocyte-derived macrophages (*P*<0.05; Fig. 2A). Ucn1 also significantly suppressed CD36 and ACAT1 protein expressions in a concentration-dependent manner (*P*<0.05, *P*<0.001; Fig. 2B, 2C). However, Ucn1 did not affect significantly ABCA1 protein expression in human monocyte-derived macrophages (*P* = NS; Fig. 2D).

### Effects of Ucn1 on Human VSMC Migration and Proliferation

**Figure 3:**
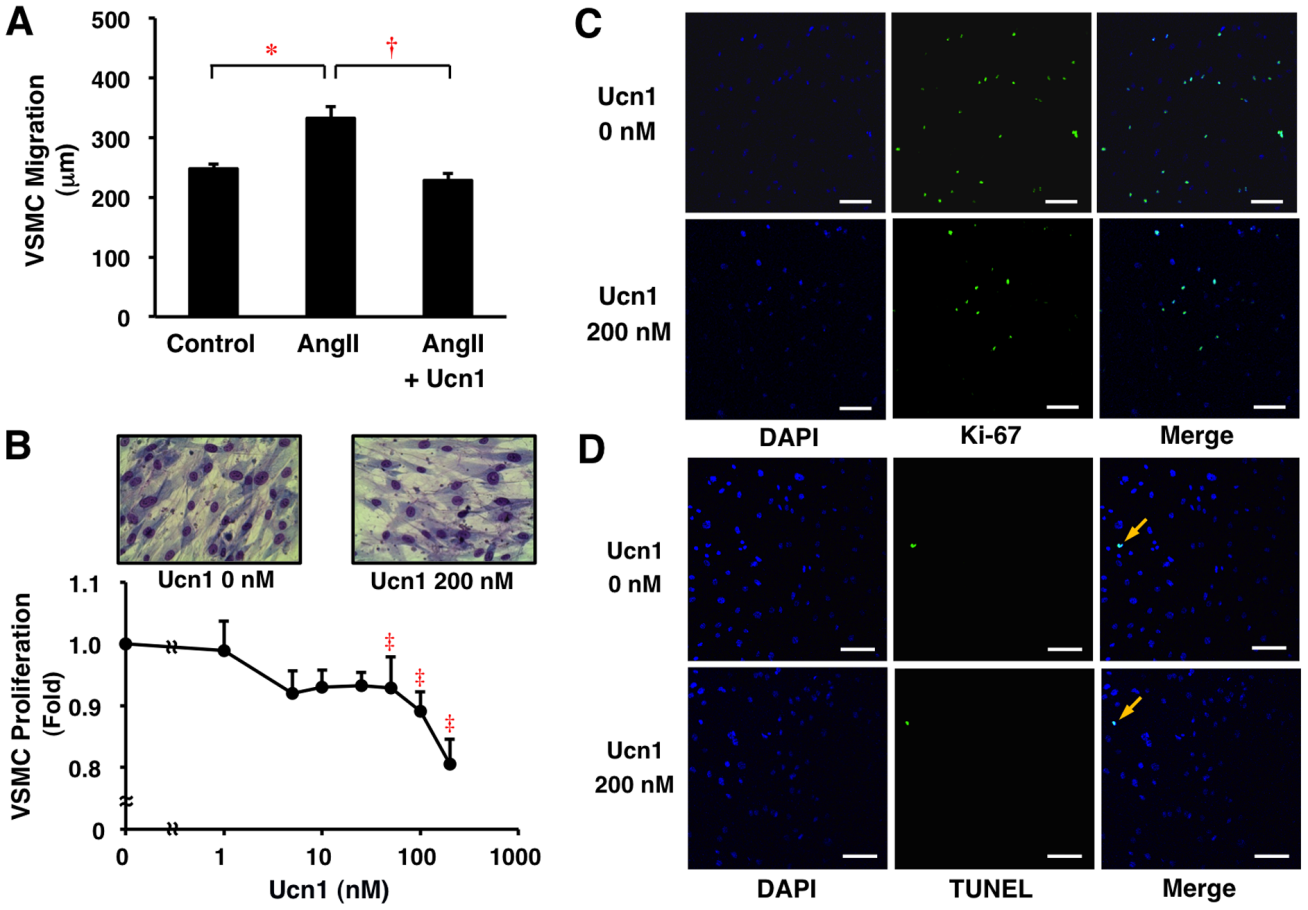
Suppressive effects of Ucn1 on migration and proliferation of human VSMCs. (A) Migration of HASMCs was determined using a BIOREVO BZ-9000 microscope culture system in serum-free SmBM without or with AngII (500 nmol/l) and/or Ucn1 (200 nmol/l) (n = 10). (B) Proliferation of HASMCs was determined by WST-8 assay after 48-h incubation in conditioning medium with the indicated concentrations of Ucn1 (n = 4). Data represent means ± SEM. *P<0.005, **^†^** *P*<0.001, **^‡^** *P*<0.05 vs. 0 nmol/l of Ucn1. (B–D) HASMCs were stained with Giemsa, anti-Ki-67 antibody, and TUNEL method. DAPI was used to stain the nucleus. Representative images are shown. Scale bar = 100 µm.

Treatment with AngII (500 nmol/l) significantly potentiated the migration of HASMCs (*P*<0.005; Fig. 3A). Ucn1 (200 nmol/l) significantly suppressed AngII-induced migration of HASMCs (*P*<0.001; Fig. 3A).

Ucn1 significantly suppressed the proliferation of HASMCs in a concentration-dependent manner, with the maximal effect observed at 200 nmol/l (*P*<0.05; Fig. 3B). The inhibitory effect of Ucn1 on HASMC proliferation was confirmed by the reductions of cell number (Fig. 3B) and Ki-67 expression (Fig. 3C). However, the induction of apoptosis in HASMCs was not observed at 200 nmol/l of Ucn1 (Fig. 3D).

### Effects of Ucn1 on ECM Expression and Activity in Human VSMCs

**Figure 4:**
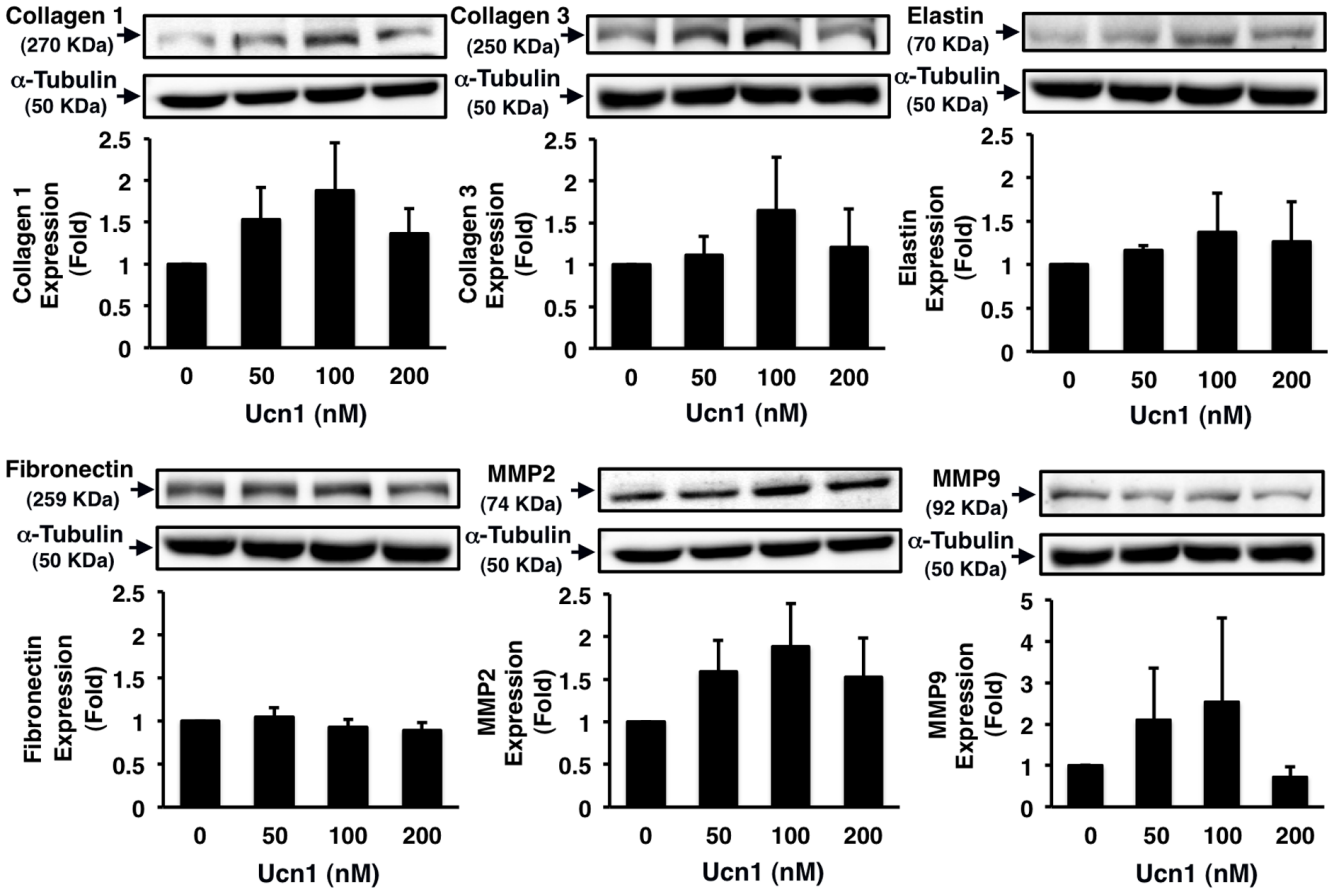
Effects of Ucn1 on ECM expression in human VSMCs. HASMCs were incubated for 24 h in serum-free conditioning medium with the indicated concentrations of Ucn1, and harvested to subject to immunoblotting analysis for collagen-1, collagen-3, elastin, fibronectin, MMP2, or MMP9. α-Tubulin served as a loading control. Data are expressed as means ± SEM from 5 independent experiments (3 independent experiments; MMP9 only).

**Figure 5:**
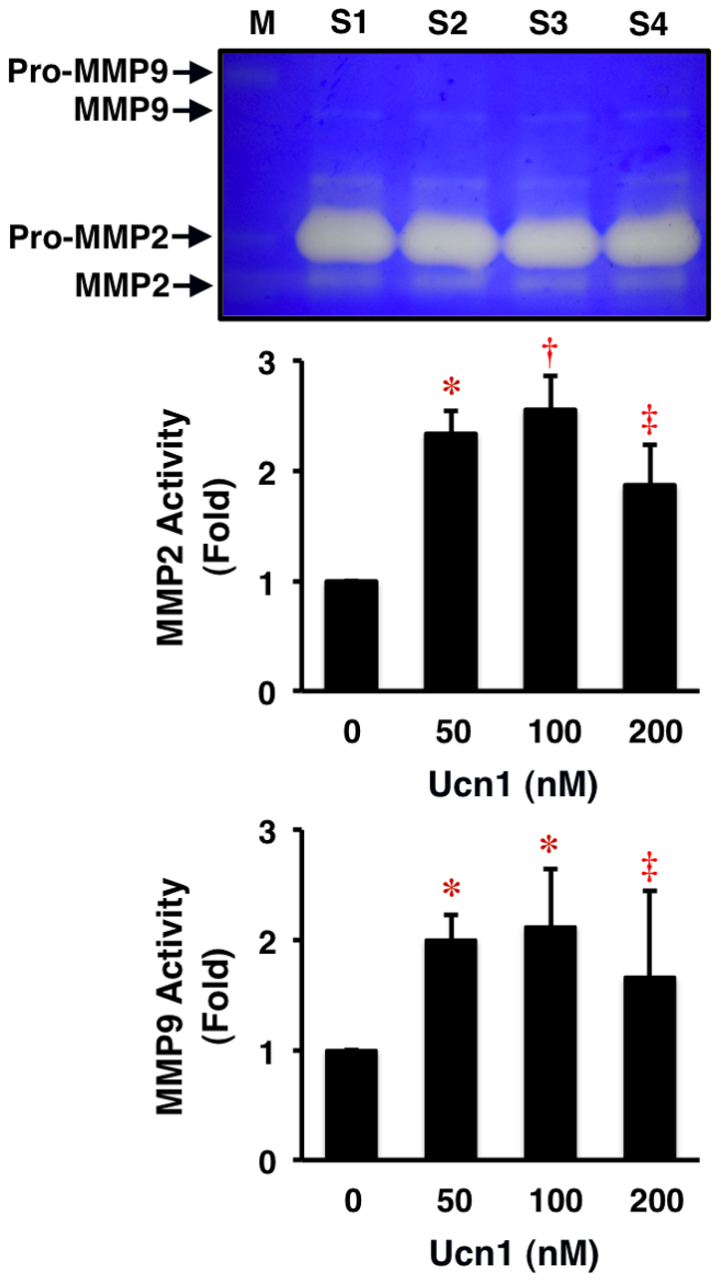
Stimulatory effects of Ucn1 on MMP2 and MMP9 activities in human VSMCs. HASMCs were incubated for 24 h in serum-free conditioning medium with the indicated concentrations of Ucn1. In the culture supernatant, MMP2 and MMP9 activities were determined by the zymography assay with 0.1% gelatin as a substrate in a 10% SDS-PAGE. Data are expressed as means ± SEM from 5 independent experiments. *P<0.005, **^†^** *P*<0.001, **^‡^** *P*<0.05 vs. 0 nmol/l of Ucn1. M = MMP marker, S = sample.

Ucn1 did not affect significantly the protein expressions of elastin and fibronectin in HASMCs. However, Ucn1 increased slightly but not significantly the protein expressions of collagen-1, collagen-3, MMP2, and MMP9 (*P* = NS; Fig. 4). Further, Ucn1 significantly enhanced the activities of MMP2 and MMP9 (at least *P*<0.05; Fig. 5).

### Effects of Ucn1 on Atherosclerotic Lesion Development in *Apoe* ^−/−^ Mice

**Figure 6:**
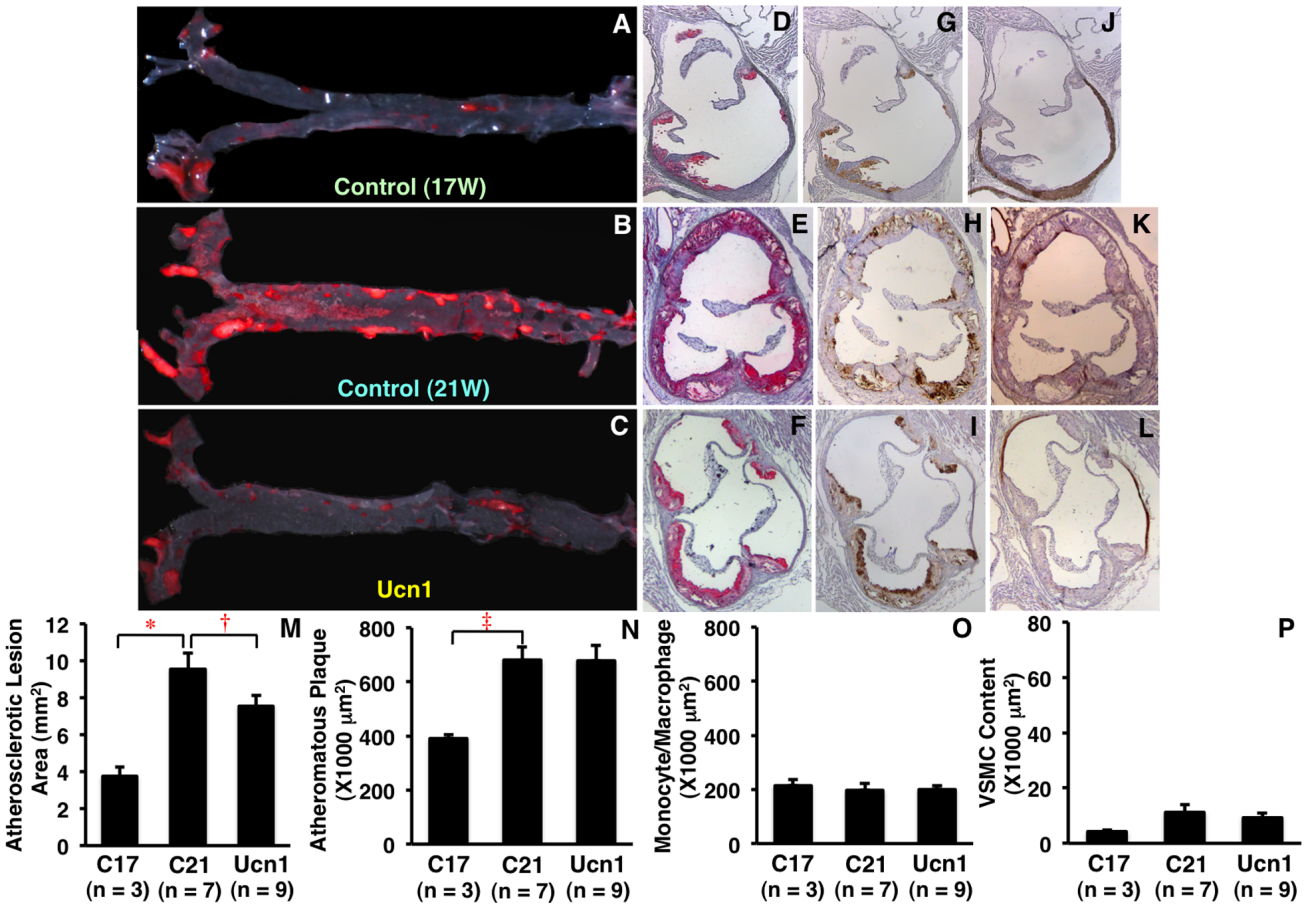
Suppressive effects of Ucn1 on the development of atherosclerotic lesions in *Apoe* ^−/−^ mice. Among 19 *Apoe^−/−^* mice at 17 weeks of age, 3 control mice were sacrificed before injection, and 7 mice and 9 mice were intraperitoneally injected for 4 weeks with saline (control) or Ucn1 (64 nmol/kg/day), respectively. The excised aortas were opened longitudinally, followed by oil red O staining (A–C). In cross-sections of the aortic sinus, atheromatous plaques (red), monocyte/macrophage infiltration (brown), or VSMC contents (reddish brown) were stained with oil red O (D–F), MOMA2 (G–I), or SMA (J–L), respectively. Hematoxylin was used to stain the nucleus. Data are expressed as means ± SEM from the indicated number of mice (M–P). *P<0.005, **^†^** *P*<0.05, **^‡^** *P*<0.01.

In *Apoe* ^−/−^ mice, aortic atherosclerotic lesions were markedly (by 2–2.5-fold) developed at 21 weeks of age compared with 17 weeks of age (Fig. 6A, 6B, 6D, 6E). Intraperitoneal injection of Ucn1 into *Apoe* ^−/−^ mice from 17 to 21 weeks of age significantly retarded the surface areas of the atherosclerotic lesions, with a significant increase in plasma Ucn1 concentration, as compared with a counterpart (Fig. 6B, 6C, 6M; Table 2). However, there were no remarkable reductions with Ucn1 injection in monocyte/macrophage infiltration and VSMC contents within atheromatous plaques in the aortic sinus (Fig. 6D–L, 6N–P). As listed in Table 2, there were no significant differences in body weight, food intake, systolic and diastolic blood pressures, or plasma concentrations of glucose and total cholesterol between the 2 groups of 21-week-old *Apoe* ^−/−^ mice.

**Table 2:** Characteristics and laboratory data in 21-week-old *Apoe* ^−/−^ mice.

	Control (n = 7)	Ucn1 (n = 9)	*P* value
Body Weight (g)	30.1±0.8	29.3±0.6	0.4307
Food Intake (g/day)	4.4±0.5	4.3±0.2	0.8521
Systolic Blood Pressure (mmHg)	120.2±5.4	123.8±4.1	0.5952
Diastolic Blood Pressure (mmHg)	81.4±10.2	92.6±8.5	0.4065
Ucn1 (pg/ml)	2.65±1.11	6.24±1.34	0.0334
Glucose (mg/dl)	168.6±13.7	175.1±12.3	0.7609
Total Cholesterol (mg/dl)	1824.3±12.9	1719.4±51.9	0.1077

Mean ± SEM.

## Discussion

This is the first demonstration that Ucn1 suppresses a part of inflammatory response (MCP1 and ICAM1) and proliferation of human ECs, human macrophage foam cell formation, and human VSMC migration and proliferation *in vitro*, and retards the total areas of atherosclerotic lesions on the aortic surface in *Apoe* ^−/−^ mice *in vivo*. The reason why Ucn1 could not suppress plaque size in the aortic sinus may be attributed to inflammatory vascular remodeling associated with Ucn1-induced up-regulations of collagens, MMPs, IL6, and E-selectin.

Our comprehensive research bridges the observation of abundant Ucn1 levels in the circulating blood in patients with coronary artery disease [14] and *in vitro* data for atheroprotective effects of Ucn1 on the vascular cells. These findings suggest that Ucn1 stimulated by inflammation and ischemia may emerge to protect the cardiovascular system [10], [11]. Ucn1 is now expected to have therapeutic potential in cardiovascular diseases [22]. In several studies [23], [24], Ucn1 was infused into healthy volunteers and patients with stable congestive heart failure to assess its pharmacokinetics. A recent study has reported that the treatment with pitavastatin, a hydroxymethylglutaryl-CoA reductase inhibitor, is effective to increase endogenous Ucn1 levels in humans [11].

Ucn1 produces the relaxation of human internal mammary artery *via* the release of endothelial nitric oxide [25]. Administration of Ucn1 into animals reduced plasma concentrations of renin, AngII, and aldosterone [26], [27], and inhibited hypertension-induced arterial remodeling (thickness) [28]. Ucn1 suppressed AngII-induced ROS generation in HUVECs [11], and LPS-induced TNFα release from human trophoblast cells [29]. Ucn1 also suppressed collagen-induced arthritis by down-regulation of inflammatory and Th1 responses and induction of regulatory T cells [30]. In contrast, several reports suggested the potential pro-atherogenic effects of Ucn1 as described below. Ucn1 stimulated IL6 expression in VSMCs and ICAM1 expression in ECs *via* cyclooxygenase-2, and promoted microvascular permeability during inflammation, MMP9 expression, and the development of vasculitis [31]–[35]. Ucn1 promoted cell proliferation and hypertrophy and collagen production in cardiac myocytes and non-myocytes [36], [37]. Cell growth and the expressions of collagens and MMPs are associated with cardiovascular tissue injury, repair, and regeneration with inflammation. The discrepancy of modulatory effects of Ucn1 on cell growth and ECM production is attributed to the difference in cell types and cardiovascular remodeling required in the individual situation. Future studies are needed to elucidate whether knockout of Ucn1 or its receptors in *Apoe* ^−/−^ mice may accelerate or suppress the development of atherosclerotic lesions.

The Ucn1 receptor CRF-R2 has been demonstrated to be present in monocytes/macrophages, ECs, VSMCs, and cardiomyocytes [5], [6], [38]. Several lines of evidence indicate that intracellular signal transductions of Ucn1-induced cardiovascular protective effects involve CRF-R2, protein kinase C (PKC), and extracellular signal-regulated kinase (ERK) 1/2 pathways [22], [39]–[41]. Cell migration is suppressed by Ucn1 *via* CRF-R2 [42]. Previous studies from our and other's groups have shown that foam cell formation and ACAT1 expression in macrophages and the migration and proliferation of VSMCs are mediated *via* PKC and ERK1/2 pathways [16], [18], [19], [43], [44]. Further studies are needed to elucidate whether all phenomena observed in our study could be mediated *via* the above pathways using the selective CRF-R2 antagonists and the individual specific inhibitors.

We discuss the integrity of Ucn1 concentrations in our i*n vitro* and *in vivo* experiments. First, the concentrations of Ucn1 required for enhancement of ACAT1 and CD36 expression and foam cell formation in macrophages were relatively high (250–500-fold) compared with plasma Ucn1 concentrations in humans: ∼0.1 nmol/l in healthy volunteers and ∼0.18 nmol/l in patients with acute myocardial infarction [14]. In the vascular wall, it is mainly ECs and macrophages that generate large amounts of Ucn1 in an autocrine/paracrine manner. Animal and clinical studies showed that local levels of other vasoactive agents, such as AngII, were increased by ∼100-fold in cardiac interstitial fluid [45], comparable to the present study. Second, the concentration of Ucn1 in human serum from healthy volunteers was 3.72 pmol/l in our study. Therefore, the 10% concentration added to culture medium for monocytes-macrophages was negligible compared with the concentrations of human recombinant Ucn1 added. Last, plasma concentrations of Ucn1 in *Apoe* ^−/−^ mice were not as high as we expected (∼2.4-fold). The following point can be given as a reason. Since blood sampling was performed 24 h after the last peritoneal injection of Ucn1, the peptide was somewhat metabolized in the circulating blood.

In conclusion, the present results suggest that Ucn1 may retard the development of atherosclerotic lesions by suppressing EC proliferation, macrophage foam cell formation, and VSMC migration and proliferation in addition to a part of inflammatory response in ECs. Thus Ucn1-based treatments using this peptide itself and/or its analogues are expected to emerge as a new line of therapy against atherosclerosis and its related diseases.
